# Predictability of intraoral scanner error for full-arch implant-supported rehabilitation

**DOI:** 10.1007/s00784-023-05011-4

**Published:** 2023-04-12

**Authors:** Francesco Zingari, Matteo Meglioli, Francesco Gallo, Guido Maria Macaluso, Sara Tagliaferri, Andrea Toffoli, Benedetta Ghezzi, Simone Lumetti

**Affiliations:** 1 Center of Dental Medicine, Department of Medicine and Surgery, University of Parma, Via Gramsci 14, 43126 Parma, Italy; 2 Department of Maxillofacial Surgery, Galeazzi Hospital, Milan, Italy; 3 Department of Biomedical, Surgical and Dental Sciences, School of Dentistry, University of Milan, Milan, Italy; 4 Department of Maxillofacial Surgery, Italian Stomatologic Institute, Milan, Italy; 5 IMEM-CNR, Parco Area delle Scienze 37/A, 43124 Parma, Italy; 6 Department of Medicine and Surgery, University of Parma, Via Gramsci 14, 43126 Parma, Italy; 7 CERT, Center of Excellence for Toxicological Research, University of Parma, Parma, Italy

**Keywords:** Implant scan body, Implant-supported prosthesis, Full-arch scan, Accuracy, Digital dentistry

## Abstract

**Objectives:**

The present study aimed to analyze the behaviors of three intraoral scanners (IOSs): evaluating the interdistance and axial inclination discrepancies in full-arch scans, predictable errors were searched.

**Materials and methods:**

Six edentulous sample models with variable numbers of dental implants were used; reference data were obtained with a coordinate-measuring machine (CMM). Each IOS (i.e., Primescan, CS3600, and Trios3) performed 10 scans per model (180 total scans). The origin of each scan body was used as a reference point to measure interdistance lengths and axial inclinations. Precision and trueness of interdistance measurements and axial inclinations were evaluated to address error predictability. Bland–Altman analysis, followed by linear regression analysis and Friedman’s test (plus Dunn’s post hoc correction), was performed to evaluate the precision and trueness.

**Results:**

Regarding interdistance, Primescan showed the best precision (mean ± SD: 0.047 ± 0.020 mm), while Trios3 underestimated the reference value more than the others (*p* < 0.001) and had the worst performance (mean ± SD: −0.079 ± 0.048 mm).

Concerning the inclination angle, Primescan and Trios3 tended to overestimate angle values, while CS3600 underestimated them. Primescan had fewer inclination angle outliers, but it tended to add 0.4–0.6° to the measurements.

**Conclusions:**

IOSs showed predictable errors: they tended to overestimate or underestimate linear measurements and axial inclinations of scan bodies, one added 0.4–0.6° to the angle inclination values. In particular, they showed heteroscedasticity, a behavior probably related to the software or the device itself.

**Clinical significance:**

IOSs showed predictable errors that could affect clinical success. When performing a scan or choosing a scanner, clinicians should clearly know their behaviors.

## Introduction

In the past decade, the applications of digital technologies in dentistry have undergone exponential growth [1]. A reliable impression, acquired with an intraoral scanner (IOS), is the first and most crucial step in all digital workflows [2]. IOS, as defined by the ISO 20896 (International Organization for Standardization), is the combination of a hand-held scanning device suited for use in the oral cavity, and computer hardware and software that outputs a numerical, three-dimensional description of scanned surfaces. An IOS must be practical, user-friendly, cost-effective, fast, powder-free, and accurate [3].

Accuracy is defined as the result of trueness and precision by the ISO 5725 (International Organization for Standardization). Trueness refers to the closeness of agreement between the arithmetic mean of a large number of test results and the true or accepted reference value. Precision refers to the closeness of agreement between repeated test results. Therefore, a precise scanner delivers consistent results after repeated scans, while a true scanner obtains a three-dimensional object rendition that closely matches the scanned object. These two features are not necessarily correlated; for example, a scanner with high (low) trueness may have low (high) precision.

The overall performance of full-arch scans in edentulous patients for implant-supported prostheses has been a topic of debate among researchers and clinicians. There is no consensus regarding whether IOSs can substitute traditional impressions for the fabrication of implant-supported prostheses in completely edentulous patients [4–10]. Instead of relying on transfer copings, IOSs retrieve the information regarding implant position, angle, and height through implant scan body (SB) [11]. When performing full-arch digital scans, SB geometry, material and position could affect final accuracy [11, 12].

Most of the literature, varying in methods and superimposition techniques [13], describes accuracy analyses in terms of absolute variability between scan bodies and implant analog interdistances [14, 15]. To our knowledge, no published studies have analyzed the relationship between the amount of error and interdistance or angle value (i.e., the predictability of IOS error).

Our research hypothesis was “Is it possible to find out predictable errors assessing intraoral scanner accuracy in full arch-scan for implant supported rehabilitations?” Thus, an in vitro study was performed, involving different IOSs, by addressing the error predictability in models with varying numbers of dental implants. We evaluated interdistances among scan bodies and their corresponding inclination angles.

## Materials and methods

### Study protocol

This in vitro study was performed to evaluate the error predictabilities of three commercial IOSs. According to the ISO 20896, that assess the accuracy evaluation of IOSs, under constant conditions, the same expert operator scanned six different plaster models made with a variable number of scan bodies. The model-reference data were acquired through a coordinate-measuring machine (CMM). The interdistances and inclinations of the scan body’s axes were calculated using the retrieved STL files. These data were compared with data obtained from the CMM.

**Fig. 1:**
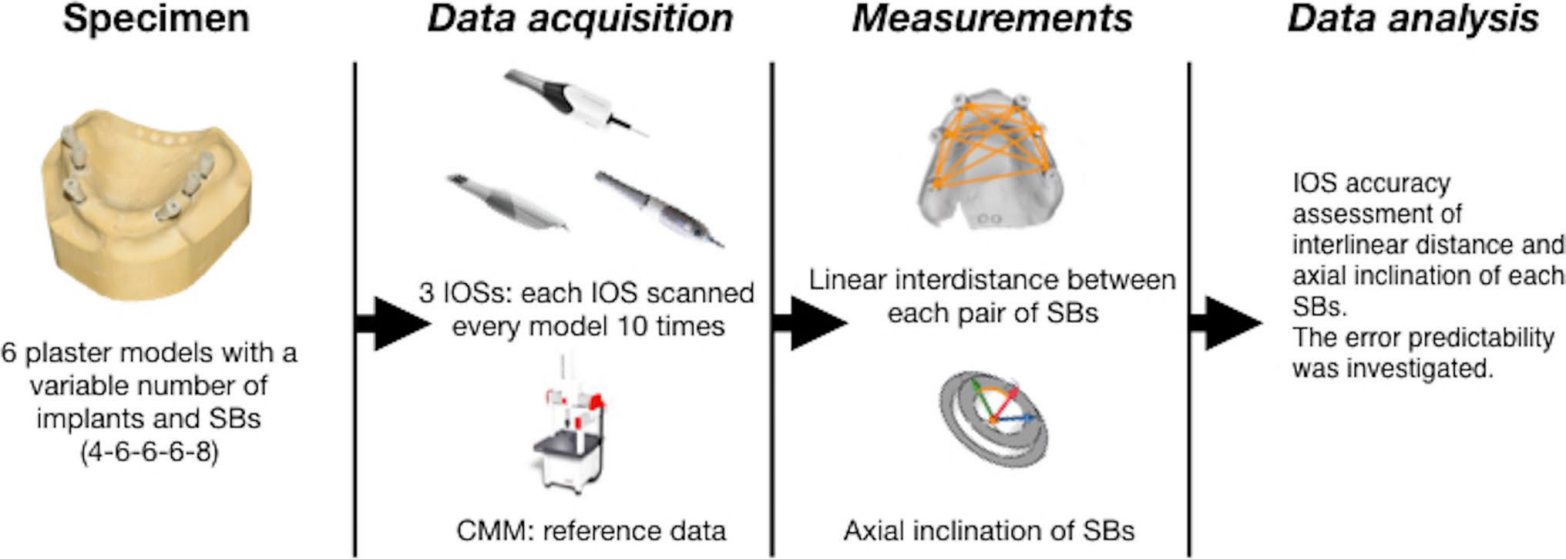
Overview of the study protocol

The precision and trueness of each scanner were analyzed via statistical tests of interdistances and axes inclinations. The mathematical relationship between these measurements, as the scan-abutment distances increased, was investigated (Fig. 1).

### Models

Six models, based on the same mold of an edentulous maxilla, were made using plaster class IV (GC Fujirock, Belgium). The operator then created holes in the models, using a core drill (diameter: 4 mm), in the middle of the bone crest. Four to eight implant analogs (Implant Way Mix; diameter: 3.8 mm; IESS Group, Italy) were placed with an unknown orientation and fixed in class IV plaster.

Four models with 6 implant analogs, one model with 4 implant analogs, and one model with 8 implant analogs were fabricated. One IESS group scan body was screwed on each implant analog. This polyether-ether ketone scan body had a titanium metallic connection, a diameter of 5 mm, and a height of 8 mm.

### CMM test

A mechanical CMM measures the geometry of an object by detecting discrete points on the surface of the object using a probe. The Coord3 CMM Benchmark, Perceptron (Coord3, Italy) was used in this study with a power-head PH10M plus, Renishaw 7.5° step 15 axis position A, 105° 48 axis position B ± −180°, sensor Ø1 mm TP20 SF, Renishaw 0.5 μm reprod., and uniaxial ± 1 μm reprod. on changing module. To acquire the analog space position data, one metallic cylinder (i.e., locator) with known dimensions was screwed on each implant analog.

After multiple contacts, the CMM registered the diameter and the plane at the top of the cylinders. With the resulting space position data and cylinder dimensions, the researchers were able to obtain the origin P_0_, which corresponded to the circle inscribed in the polygon at the base of the analog. Thus, P_0_ represented the spatial position of the analog on 3 axes (x, y, and z). The CMM was capable of a three-dimensional maximum error assessed as E3-xyz (L) = 2.8 + 5 L/1000 mm (where L is the measured distance in millimeters, according to ISO 10360 standard). Therefore, the CMM data were regarded as true data.

### Digital impressions

In this study, three different intraoral scanners were used: CS3600 version 3.1.0 (Carestream Dental, USA), Trios3 (3shape, Denmark), and Cerec Primescan version 5.1.3 (Dentsply Sirona, USA). Each model was scanned ten times by the same expert operator, in accordance with the manufacturer’s instructions, and under the same conditions (21 °C and 80% humidity). The operator waited 10 min between scans. Each scanner performed 60 digital impressions.

### Scanning strategy

The operator employed the same scanning strategy with all the IOSs. At the adequate scan high, the most distal scan body was scanned on the upper side, followed by the buccal and the palatal ones. Subsequently, the mucosa between the scan body detected and the following one was scanned without interrupting the scan flow at any moment. Then, the reached scan body was scanned on each side. The operator scanned all the scan bodies in continuous flow. Scanning defects were fixed once the full-arch scan was acquired.

### STL-file processing

**Fig. 2:**
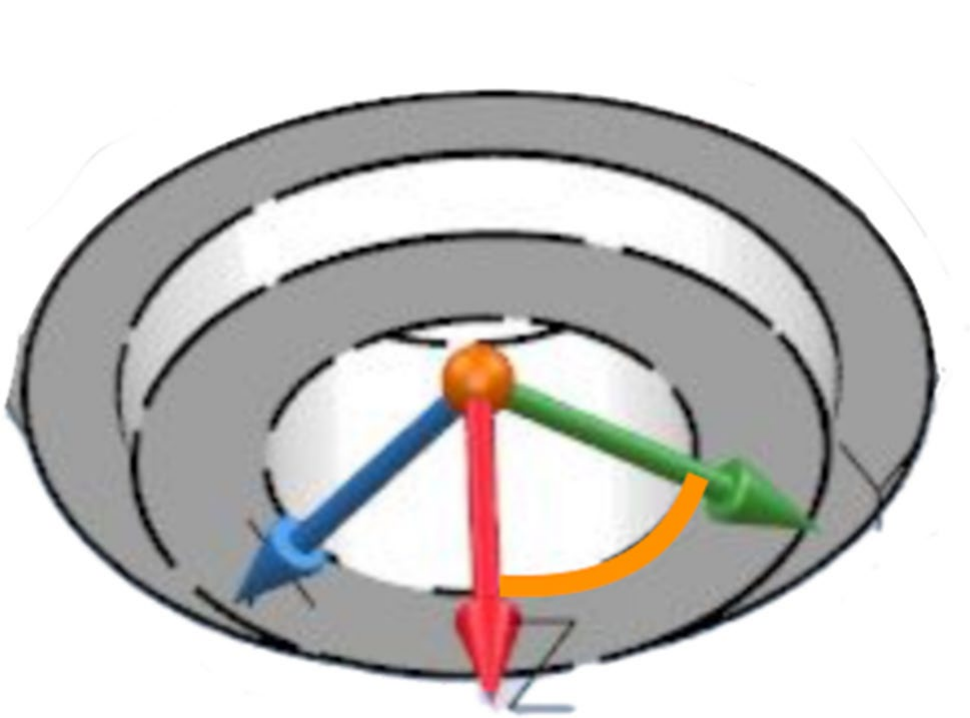
P_0_: the point that represents the spatial coordinates of the SB platform

To derive P0 from digital impressions, scan files were imported on Exocad (Exocad GmbH, Germany), a dental computer-aided design software. The STL files were opened, and scan bodies, registered with the IOSs, were superimposed on standard scan bodies present in the Exocad library. These two scan bodies had been aligned using the “best-fit” algorithm. Following the matching, knowing the mathematical quotes of each library scan body, the P_0_ of scan body platforms were obtained. P0, representing the spatial coordinates of the analog platform (Fig. 2), was extracted both from the STL file and the models through the CMM. Thank to P0 was possible to analyze discrepancies between measurements retrieved through IOSs and the CMM.

### Interdistance measurements

**Fig. 3:**
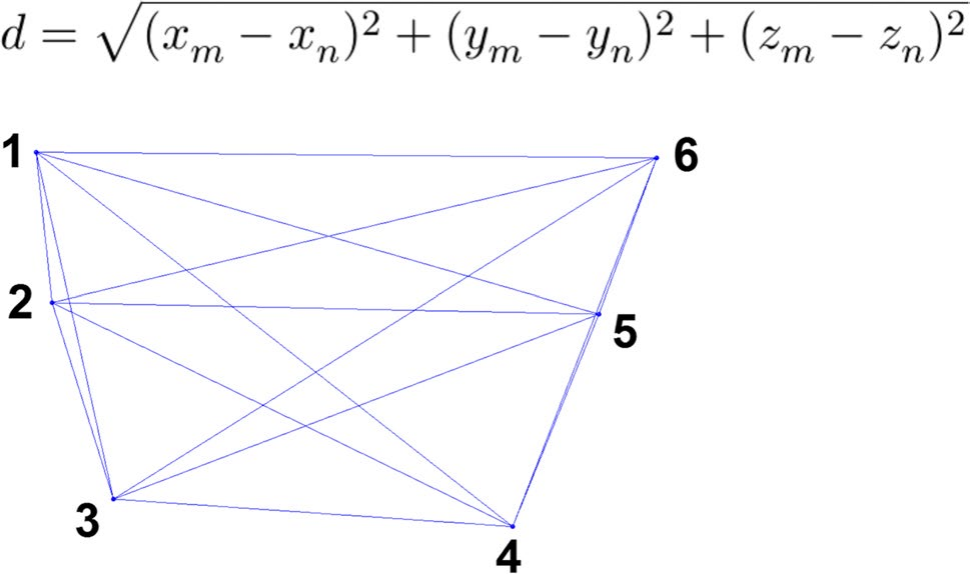
Formula and scheme used to analyze the interdistance measurements

P_0_, the origin point of each analog, was used to measure the distance between each pair of analogs. This point was represented by three axial coordinates (x, y, and z); distances were calculated using the formula below. The distance of every possible analog pair was considered, as shown in Fig. 3. Distances evaluated on the IOS data were compared with distances obtained from the CMM. Both precision and trueness were determined via statistical analyses.

### Axial inclination measurements

**Fig. 4:**
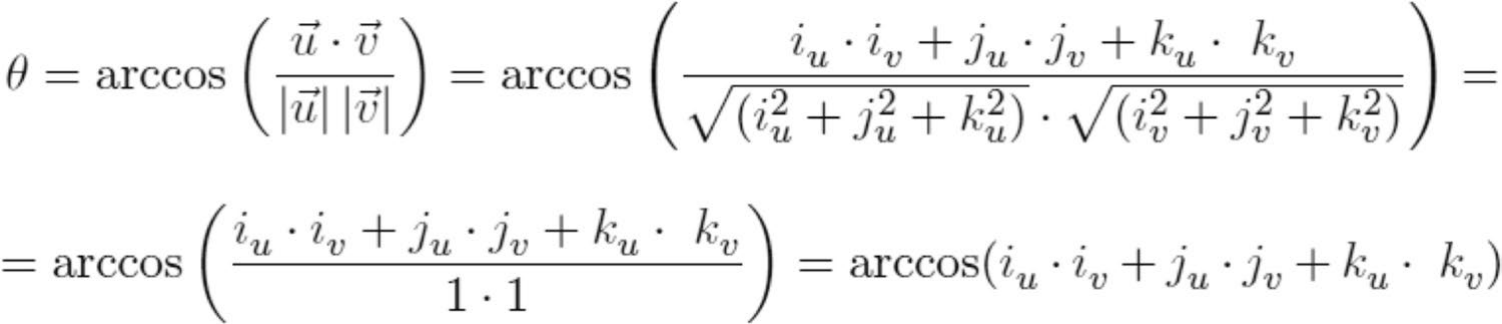
Formula used to analyze the axial inclination

Errors in the axial inclinations of the scan bodies were calculated with P_0_ as the origin. Each origin measurement was made of three vectors (i, j, and k); angles between each couple of scan body origins were calculated using the formula below (Figure 4). Each vector has three components in the Cartesian planes (xy, yz, and yz), physically measuring the angle between two distinct vectors. So, it was possible to find the plane in common and calculate the angle on this plane and to evaluate the misalignment between the axes of the scan body.

Eventually, the retrieved angles were compared with angles obtained from the CMM. Both precision and trueness were determined via statistical analyses.

### Statistical analysis

The term “accuracy,” as defined by ISO standard 5725-1, encompasses both trueness and precision. “Trueness” is the measured deviation from the actual value or dimension of the object. “Precision” is a measure of repeatability or how close a set of results are to each other. Evaluating the precision means to analyze the error variability among replicates: the more precise a device is, the less error variability it shows.

Precision has been calculated as the standard deviation of replicates (*n* = 10; i.e., scans) of each interdistance or axial inclination measured by all pairs of scan bodies (*n* = 15 values for models 1, 2, 4, and 6; *n* = 28 values for model 3; and *n* = 6 values for model 5, for 94 total values). The calculation was performed considering all values together and then stratified by model. Trueness was calculated as the difference between the mean value of 10 scans and the value obtained by CMM, for each combination of pairs of scan bodies, both for interdistance and axial inclination. Also in this case, 94 total values for each IOS were used and then stratified by model.

Bland–Altman analyses were performed to investigate precision and trueness [16]. These analyses were performed for the interdistances and the axial inclinations of the scan bodies. Linear regression analyses were performed using Pearson’s *r*^2^ to investigate IOS precision in terms of interdistances and axial inclinations. The distributions of trueness deviations were also reported.

Because of deviations from normality in the variability data, comparisons among IOSs were performed using Friedman’s test followed by Dunn’s post hoc test. Finally, the overall deviation from zero was tested by using the hypothesis test on a mean, with zero regarded as the null hypothesis.

A mixed model was used to analyze the deviation from reference values (defined as the difference between values of 10 scans and real measure for each combination of pairs of scan bodies), with model and type of IOS as between factors and scans as repeated measure (within factor). If Mauchly’s sphericity assumption was met, Mauchly’s test was used, whereas if Mauchly’s sphericity assumption was not met, the Huynh-Feldt test was used.

A *p-*value < 0.05 was considered statistically significant. All Bland–Altman analyses were performed using Origin Pro 2021 (OriginLab Corp., Northampton, MA, USA), while statistical comparisons were performed using GraphPad Prism 8.0 (GraphPad, San Diego, CA, USA).

## Results

### Precision evaluation of linear interdistances

**Fig. 5:**
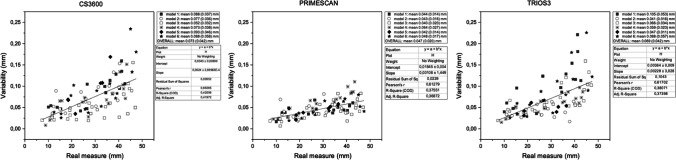
Graphical representation of precision analysis for the interdistance measurements

As previously affirmed, assessing the precision means to analyze the error variability. Thus, the graphs show *variability* on the *x* axes. The statistical results for IOS linear variability are shown in Fig. 5. Primescan showed the best performance (mean: 0.047 mm; standard deviation: 0.020 mm), followed by Trios3 (mean: 0.069 mm; standard deviation: 0.042 mm) and CS3600 (mean: 0.073 mm; standard deviation: 0.042 mm).

For all IOSs, error variability increased with distance. As heteroscedasticity was observed, we performed a linear mixed model with model and type of IOS as between factors and scans as repeated measure (within factor). This approach revealed significant scan, IOS, scan*model, scan*IOS, model*IOS, and scan*model*IOS effects (*p* < 0.001 for all factors), while the model did not show a significant effect (*p* = 0.857). Scan and IOS are significant factors affecting the precision of three IOSs, and interestingly, their behaviors appear different from each other.

**Fig. 6:**
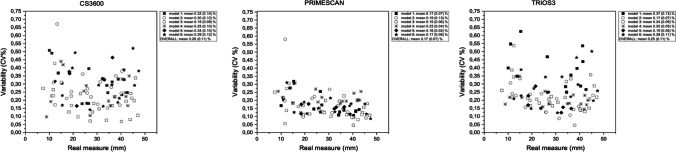
Absolute error transformed into percentage relative error

When the absolute variability was transformed into the coefficient of variation as a percentage (CV%, defined as variability_IOS_/real measure_CMM_*100), the resulting percentage relative error generally remained constant among measurements (Fig. 6).

### Trueness evaluation of linear interdistances

**Fig. 7:**
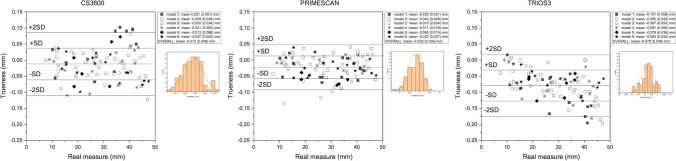
Graphical representation of trueness analysis for the interdistance measurements

The results about IOS trueness are presented in Fig. 7, showing the Bland–Altman plots. For CS3600, we observed a trueness with a mean of −0.012 mm and a standard deviation of 0.049 mm. Both devices, CS3600 and Primescan, tended to underestimate the real measure of linear interdistance (*p* = 0.018, *p* < 0.001, respectively). Trios3 showed underestimation of the real measure (*p* < 0.001), which increased with increasing distance for Trios3. This device exhibited the worst trueness (mean: −0.079 mm; standard deviation: 0.048 mm).

### Linear interdistance: comparison among IOSs

**Fig. 8:**
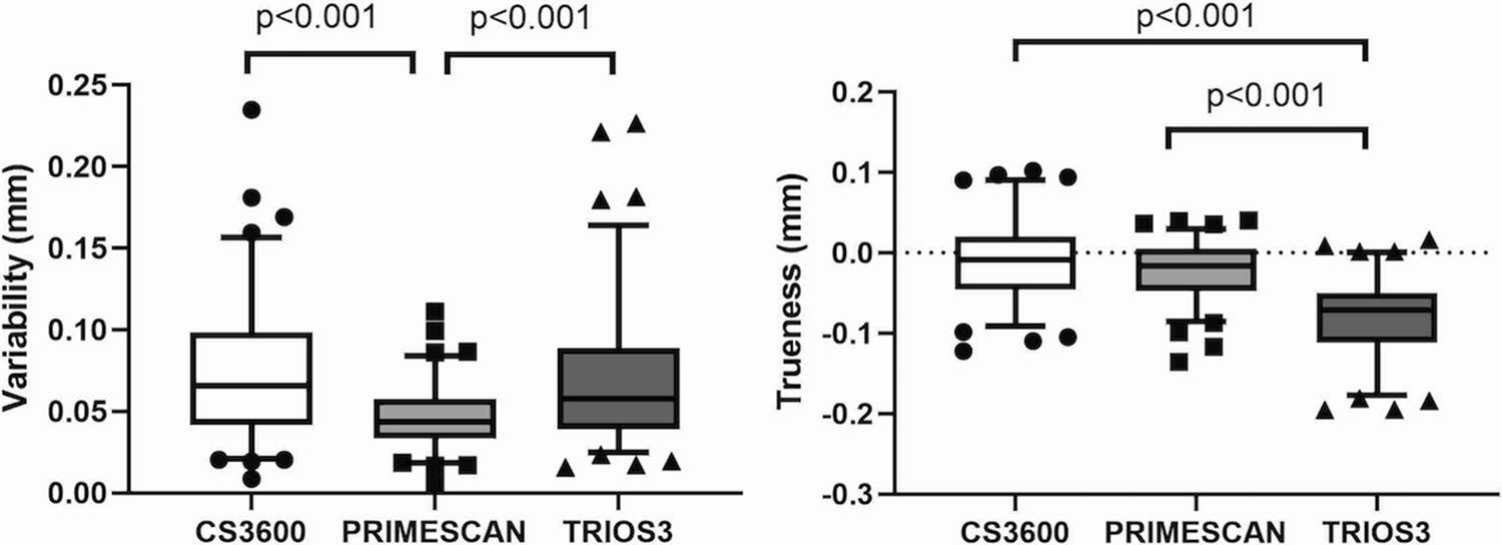
Absolute linear discrepancy comparison among IOSs in terms of precision and trueness. Solid black line represents the median value. Top and bottom of the box represent the 75th and 25th percentiles, respectively. Whiskers represent the maximum and minimum values, while geometric figures represent the outliers

Comparisons of variability and trueness are shown in Fig. 8. The precisions of Trios3 and CS3600 were similar, while Primescan showed fewer outliers and less variability. Regarding trueness, Trios3 underestimated measurements, compared with the other scanners; CS3600 and Primescan exhibited similar findings (Fig. 8).

As suggested by the linear mixed model previously mentioned, scan and IOS are significant factors affecting the accuracy of the three IOSs, and interestingly, their behaviors appear different from each other.

### Precision evaluation of axial inclination angles

**Fig. 9:**
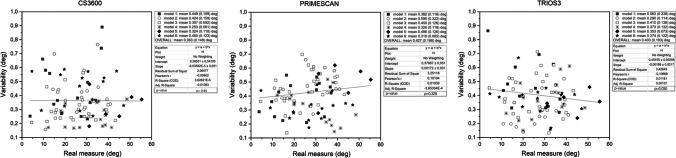
Graphical representation of precision analysis of the axial inclination of SBs

The analysis of precision (variability) in axial inclination angle is depicted in Fig. 9. Precision was not influenced by increasing angle. Primescan showed fewer outliers, compared with CS3600 and Trios3 (Fig. 9).

### Trueness evaluation of axial inclination angle

**Fig. 10:**
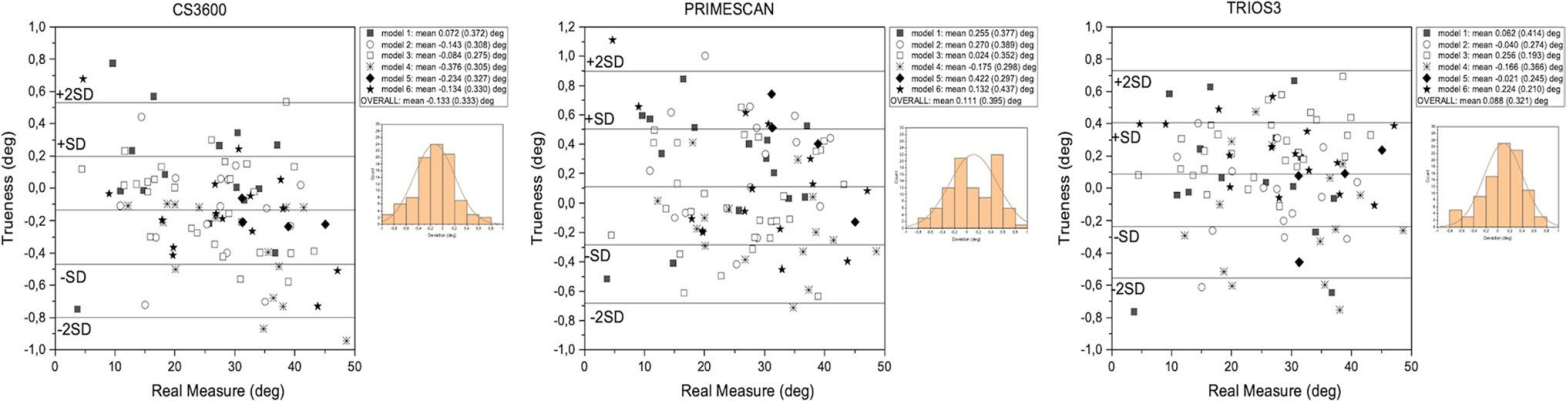
Graphical representation of trueness analysis of the axial inclination of SBs

The trueness evaluation of axial inclination angle for the three scanners and the Bland–Altman plots are presented in Fig. 10.

The analysis on CS3600 showed that some cases were not included in the confidence interval and suggesting deviation from congruency between the scanner and the CMM (*p* > 0.001). A wider angle was related with greater underestimation by CS3600. In contrast, Primescan slightly overestimated the angles (*p* = 0.008). The residuals distribution of Primescan tended to be bimodal, with an irregular tendence to add 0.4° and 0.6°. This could explain the tendency towards overestimation. Trios3 tended to overall slightly overestimate the angles (*p* = 0.01). Left asymmetry was present in the distribution, indicating that underestimation could affect important values. The residuals did not increase with increasing angle (Fig. 10).

### Axial inclination angle: comparison among IOSs

**Fig. 11:**
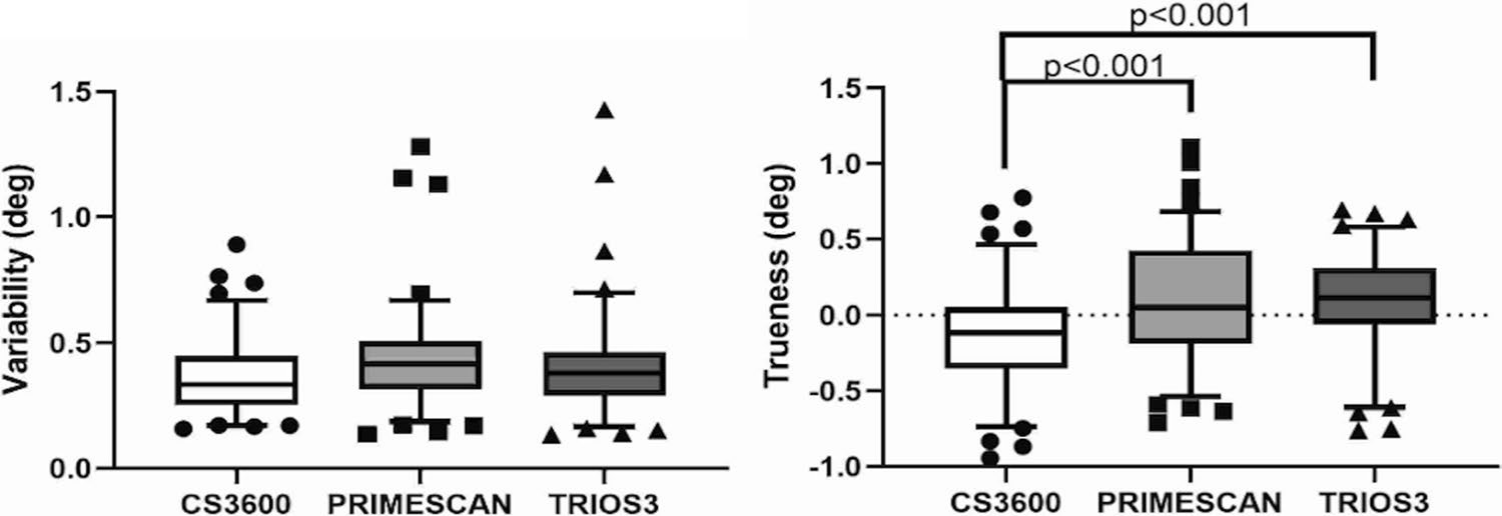
Axial inclination comparison among IOSs in terms of precision and trueness. Solid black line represents the median value. Top and bottom of the box represent the 75th and the 25th percentiles, respectively. Whiskers represent the maximum and minimum values, while geometric figures represent the outliers

There were no significant differences in inclination angle variability among IOSs (Fig. 11). Regarding trueness, CS3600 tended to underestimate, while the other two scanners overestimated the values (Fig. 11).

The analysis of variance of axial inclination data showed a significant effect of scan (*p* = 0.002), model (*p* < 0.001), IOS (*p* < 0.001), and their interactions (*p* < 0.001 for scan*model, scan*IOS, model*IOS, and scan*model*IOS).

## Discussion

The main findings of the present study revealed that Primescan, CS3600, and Trios3 showed predictable errors in assessing intraoral scanner accuracy in full-arch scan for implant-supported rehabilitations. In particular, they tended to overestimate or underestimate linear measurements and axial inclinations of scan bodies, one added about 0.4–0.6° to the angle inclination values. Furthermore, we observed a heteroscedastic behavior probably related to the software or the device itself.

Accuracy is crucial for the fabrication of implant-supported prostheses because prosthetic misfits lead to clinical failure. Many authors have investigated the accuracies of IOSs for full-arch scans to determine whether they could replace conventional impressions [4, 6, 17, 18]. Several studies have shown conflicting results regarding the accuracies of scanners employed in implant-prosthetic workflows. This suggests that some IOSs are unsuitable for full-arch digital impressions [17]. The acceptable level of inaccuracy ranges from 50 to 150 μm [21, 22].

Unfortunately, it is difficult to compare these studies because of differences in measurement methods, IOS versions, scan body shapes and sizes, materials, and sample types [20, 23]. Some studies have involved dentate models [4, 17, 24, 25], while others have used edentulous samples [10, 26–28]. Furthermore, the methodology for evaluating accuracy differed among studies. Reference measurements obtained using CMMs are preferable, compared with measurements obtained using industrial scanners [20]. Nevertheless, it is important to process the meshes to identify unique scan-abutment points through the original computer-aided design files used to produce the abutments [19]. Many authors have used computer-aided design software with best-fit algorithms to perform mesh-to-mesh alignment [6, 15, 25, 29–31]. From a metrological perspective, the accuracy estimate obtained using this methodology is unacceptable for the assessment of IOS performance according to ISO 10360 standards [20]. In addition, operator experience [2], powder use [32], and the scan body [11] and sample materials [33] can influence the results. ISO20896 is the standard that defines test methods and procedures for assessing IOS accuracy. According to this document, the models were fabricated with plaster class IV, more than 30 scans for every IOS (i.e., 60 scans/IOS) were performed, and SBs with an adequate dimension (ø5 ± 0.01 mm) were screwed on implant analogs.

In addition, the test models showed a suitable minimum number of reference objects (i.e., 4-6-8 SBs). Despite the efforts to follow ISO20896, the presented method differs from the ones involved in ISO 20896. SBs, that give the possibility to investigate axial inclination, were used instead of the spheres; the statistic goes beyond the ISO20896 one, considering trueness and precision separately; and CMM measurements were regarded as true data.

According to the previous consideration, we prefer not to compare our results with previous findings.

The purpose of this study was to analyze the behaviors of these devices, focusing on error predictability.

Multiple predictable behaviors were found: a tendency to overestimate or underestimate the linear measurements, heteroscedasticity in analyses of linear variability when scanning longer spans, and a tendency to add 0.4–0.6° in the axial inclination. These errors may have been caused by the software or the device itself. Regarding interlinear distances, Primescan was the most precise scanner, while CS3600 had the best trueness. Primescan showed fewer outliers and had a tendency towards underestimation. The tendency for underestimating linear measurements was present in all IOSs, although it was greater in Trios3 than in CS3600.

Focusing on interdistances measurements’ precision, all the intraoral scanners showed heteroscedasticity, even if the variability error transformed into relative variability (CV%) showed different behavior with the tendency of variability to be constant for increasing distances. Regarding Trios3 and CS3600 results, variability increased in absolute volume with increasing measurement values. However, the percentage error remained constant. Thus, accuracy was dependent on a coefficient linked to the instrument, which is equally important to the distance itself. On the other hand, the Primescan percentage relative error tended to slightly decrease for increasing distances.

A linear mixed model was performed, and the variance components of different scanning replicates (i.e., scans), models, IOSs, and their interaction terms were analyzed. Regarding the interdistances, the mixed model revealed that IOS and scans significantly affect the trueness of measurements (*p* < 0.001). Furthermore, a significant effect was described for scan*model, scan*IOS, model*IOS, and scan*model*IOS interaction terms (*p* < 0.001 for all factors), showing different behaviors of scans, models, and IOSs on measurement. On the other hand, we did not observe a significant effect of the model (*p* = 0.857).

The analysis of variance of axial inclination data showed a significant effect of scan (*p* = 0.002), model (*p* < 0.001), IOS (*p* < 0.001), and their interactions (scan*model, scan*IOS, model*IOS, and scan*model*IOS: *p* < 0.001 for all factors). It can be inferred that scan, model, and IOS are significant factors that affect the accuracy of IOSs. Researchers and IOSs manufacturers should investigate the correlation between variance components because they showed different behaviors and impact on the final result. Further studies are necessary to understand whether by modifying a variance component might lead to an enhancement of performance in terms of resulting accuracy.

Regarding the axial inclination angle, none of the IOSs showed a heteroscedastic behavior. Primescan performed better than did Trios3 or CS3600, although it tended to add 0.4–0.6° to the measurements. Primescan bimodal distribution of residuals was unique. Moreover, CS3600 underestimated in proportion to the angle, while Trios3 overestimated the values by a constant amount.

Precision decreased with longer distances presumably because of the stitching process [34]. Scanning errors associated with image stitching tend to accumulate when the scanned objects are flat or planar, such as a residual ridge [35]. Trueness was reported to improve when scanning aids were used [36]. Miyoshi et al. suggested that digital impressions for implant treatment should be limited to small prostheses, such as a 3-unit superstructure supported by two implants [26]. Kernen et al. showed that intraoral scanning resulted in clinically unacceptable accuracy for long-span virtual models [37]. However, several authors have reported the superiority of digital impressions, compared with conventional impressions. Pesce et al. reported successful clinical results using intraoral scanners and scanning powder [38]. The present study used scan bodies in polyether-ether ketone that showed the best performance in previous literature [27, 33].

Most studies thus far have not considered various factors that can affect intraoral scans, such as the presence of saliva, light conditions, soft and hard tissue reflections, humidity, intermittent acquisition, and movement of the soft tissue and tongue [23, 39, 40]. In contrast to these factors, the employed type IV plaster models are optimal material for scanning. Indeed, reflective material can have a dramatic impact in scan accuracy [33]. Mucosal displacement achieved by conventional impressions cannot be replicated by IOSs [41]. Further in vivo studies are necessary to explore the applications of IOSs in implant treatment and to determine whether IOSs have clinically acceptable accuracy for the fabrication of implant-supported prostheses.

## Conclusions

Based on the findings of this in vitro study regarding IOSs, the following conclusions were drawn:

- All the IOSs showed a heteroscedastic behavior when variability was evaluated: scanners’ precision decreased in proportion to measure length. Thus, considering the percentage relative error, the variability of interdistance measurements tended to be constant for increasing distance. The analysis of trueness showed for all IOSs a tendency to underestimate the interdistance measurements.
- Overall, scans, models, IOSs, and their interactions are significant factors affecting the variability of the enrolled scanners. Surprisingly, the number of scan bodies does not significantly impact the precision of interdistance measurement.
- Primescan and Trios3 tended to overestimate the inclination angles, while angles measured by CS3600 are overall underestimated. Primescan showed a bimodal distribution of residuals showing an overestimation of 0.4–0.6° to the angle measurement.
